# Efficacy of light-cured luting systems for CAD-CAM milled resin indirect restorations

**DOI:** 10.21142/2523-2754-1403-2026-296

**Published:** 2026-07-10

**Authors:** Janeth Sagrario Quintanilla-Jiménez, Jorge Alonso Borda-Bizaga, Eliam Cielo Zúñiga-Mayhua, María Soledad Peñaherrera Manosalvas, Cristian Fernando Sánchez-Puetate, Wilfredo Gustavo Escalante-Otárola

**Affiliations:** 1 Faculty of Health Sciences, Graduate Program in Dentistry, Universidad de los Hemisferios. Quito, Ecuador. janethquintanillajimenez@gmail.com , mspenaherreram@uhemisferios.edu.ec, cfsanchezp@uhemisferios.edu.ec Universidad de Los Hemisferios Faculty of Health Sciences Graduate Program in Dentistry Universidad de los Hemisferios Quito Ecuador janethquintanillajimenez@gmail.com mspenaherreram@uhemisferios.edu.ec cfsanchezp@uhemisferios.edu.ec; 2 School of Dentistry, Universidad Católica de Santa María. Arequipa, Peru. jorgebordab@outlook.com, eliam.cielo@gmail.com Universidad Católica de Santa María School of Dentistry Universidad Católica de Santa María Arequipa Peru jorgebordab@outlook.com eliam.cielo@gmail.com

**Keywords:** dental cementation, bond strength, dental materials, adhesive dentistry, shear bond test, cementado dental, resistencia de unión, materiales dentales, adhesión dental, ensayo de cizallamiento

## Abstract

**Objective:**

To evaluate the bond strength of three light-cured luting systems for CAD-CAM milled resin indirect restorations using a shear bond strength test.

**Materials and Methods:**

Thirty Brava Block resin cylinders were fabricated using a CAD-CAM milling unit and subjected to surface treatment with 50-μm aluminum oxide airborne-particle abrasion, ultrasonic cleaning, and application of Monobond N silane primer. Specimens were randomly assigned to three groups (Variolink Esthetic LC, RelyX Veneer, and Allcem Veneer APS) and luted according to each manufacturer’s instructions. After light activation, samples were stored at 37°C for 24 hours. Shear bond strength testing was performed, and failure modes were classified. Data were analyzed using ANOVA and Tukey’s post hoc test (α = 0.05).

**Results:**

RelyX Veneer and Allcem Veneer APS protocols produced the highest bond strength values to CAD-CAM milled Brava Block resin (p < 0.05), with no significant difference between them (p > 0.05). Variolink Esthetic LC showed significantly lower values (p < 0.05). Cohesive failure within the milled resin predominated across all groups, while Variolink Esthetic LC exhibited a more balanced failure distribution (30% adhesive, 40% cohesive in resin, 30% mixed).

**Conclusions:**

The light-cured luting systems RelyX Veneer and Allcem Veneer APS provided superior bond strength to CAD-CAM milled resin compared with Variolink Esthetic LC, supporting their clinical use in indirect adhesive restorations.

## RESUMEN

**Objetivo:**

Evaluar la resistencia de unión de tres sistemas de cementación fotopolimerizables para restauraciones indirectas de resina fresada CAD-CAM, por medio de un test de cizallamiento.

**Metodología:**

Se prepararon 30 cilindros de resina Brava Block utilizando CAD-CAM, con tratamiento superficial de óxido de aluminio, baño ultrasónico y aplicación de imprimador Monobond N. Los cilindros fueron distribuidos en tres grupos (Variolink Esthetic LC, RelyX Veneer y Allcem Veneer APS) y se les aplicó el cemento correspondiente. Luego, se fotoactivaron y almacenaron a 37 °C durante 24 horas. Se realizó un ensayo de cizallamiento para medir la resistencia de unión y se clasificaron los tipos de fractura. Los datos se analizaron mediante ANOVA y Tukey, con un nivel de significancia del 5%.

**Resultados:**

Los protocolos de cementación con RelyX Veneer y Allcem Veneer APS alcanzaron los mayores valores de resistencia de unión con la resina fresada Brava Block (p < 0.05), sin diferencias significativas entre ellos (p > 0,05). Por otro lado, Variolink Esthetic LC obtuvo los valores más bajos (p < 0,05). Además, la fractura cohesiva en la resina fresada predominó en todos los grupos, y la distribución de fracturas fue más equitativa en el grupo de Variolink Esthetic LC (30% adhesiva, 40% cohesiva en resina y 30% mixta).

**Conclusiones:**

Los protocolos de cementación fotopolimerizables RelyX Veneer y Allcem Veneer APS ofrecen una resistencia de unión superior en la resina fresada mediante el sistema CAD-CAM, en comparación con el protocolo Variolink Esthetic LC.

## INTRODUCTION

CAD-CAM milled resin composites have become an important category of restorative materials due to their machinability, repairability, and favorable esthetic performance ^1^^,^^2^. These materials are commonly used for indirect restorations such as veneers, inlays, onlays, crowns, and overlays. The CAD-CAM workflow enables the fabrication of restorations with high precision and consistency ^3^, while the resin-based composition provides an attractive alternative to ceramics by offering improved elasticity, ease of adjustment, and intraoral reparability ^4^^,^^5^. Despite these advantages, achieving a reliable adhesive interface between milled resin materials and luting agents remains a clinical challenge.

Bond strength is a critical determinant of restoration longevity. It is influenced by several factors, including the intrinsic properties of the restorative material ^6^, the surface pretreatment employed ^7^^)^ and the chemical compatibility between restorative substrates and resin cements ^8^. Additionally, the thickness and translucency of CAD-CAM materials significantly affect the extent of light transmission and, consequently, the degree of polymerization of resin cements ^9^^,^^10^. Effective surface treatments-such as airborne-particle abrasion and the use of silane-containing primers-play a key role in improving adhesion to CAD-CAM resin blocks ^6^, emphasizing the need for well-defined cementation protocols that ensure a durable adhesive interface.

Light-cured luting agents have gained attention due to their improved mechanical performance, including higher tensile strength and reduced polymerization stress, both essential for maintaining restoration integrity ^11^^,^^12^. Resin cements formulated with Bis-GMA/TEGDMA matrices generally exhibit high degrees of conversion and enhanced rigidity but may be susceptible to polymerization shrinkage, which can jeopardize the adhesive interface ^13^. The incorporation of novel photoinitiators has further enhanced the polymerization kinetics and mechanical properties of these materials, offering alternatives to traditional camphorquinone-based systems ^14^^,^^15^.

Although CAD-CAM milled resin blocks offer advantages in terms of precision and mechanical behavior, adhesion-related issues necessitate a deeper understanding of both material properties and cementation strategies. Therefore, this study aimed to evaluate the bond strength of three light-cured luting systems to CAD-CAM milled resin using a shear bond strength test. The null hypothesis stated that no significant differences would be observed among the evaluated cementation protocols.

By comparing these protocols, the present study seeks to identify the most effective light-cured luting systems for optimizing adhesion to CAD-CAM resin materials, thereby providing experimental evidence to support clinical decision-making and improve the predictability and long-term success of indirect adhesive restorations.

## MATERIALS AND METHODS

### Specimen Preparation

Thirty cylindrical specimens of CAD-CAM milled Brava Block resin (FGM; Joinville, SC, Brazil) were fabricated using the DWX-42W milling system (DGSHAPE Corporation; Hamamatsu, Japan). Each specimen measured 12 mm in diameter and 14 mm in height. One circular surface of each cylinder was subjected to airborne-particle abrasion with 50-µm aluminum oxide (Bio-Art; São Carlos, SP, Brazil) for 15 seconds at 2 bar of pressure from a distance of 4 mm. The specimens were then ultrasonically cleaned for 480 seconds using a CD-4820 ultrasonic bath (Codyson Ultrasonic; Shenzhen, China) to remove any residual particles and were subsequently cleaned with 70% alcohol. A silane-based primer (Monobond N; Ivoclar Vivadent; Schaan, Liechtenstein) was applied to the treated surface for 60 seconds and dried with gentle air to eliminate excess material.

### Experimental groups

**Figure. 1:**
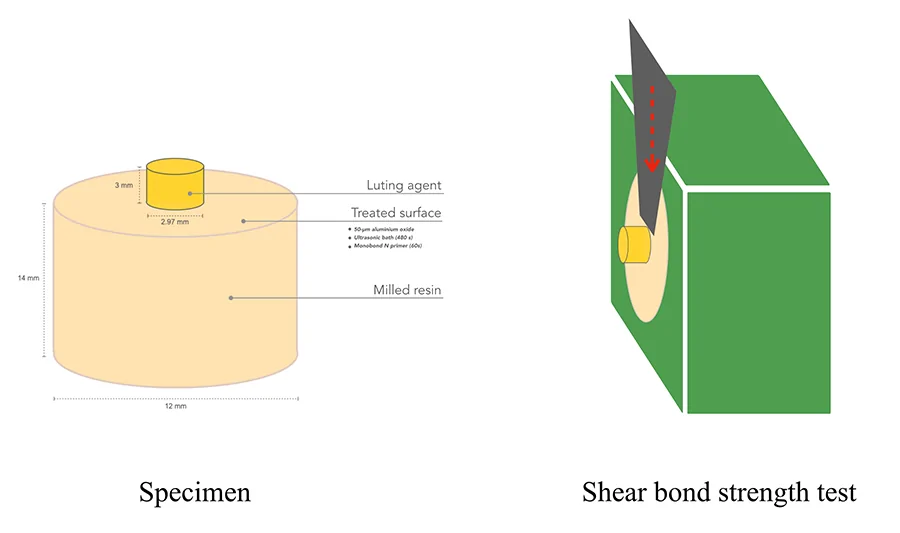
Schematic representation of the methodological workflow for specimen preparation.

The specimens were randomly assigned to three experimental groups (n = 10) according to the light-cured resin cement used: Variolink Esthetic LC, RelyX Veneer, and Allcem Veneer APS. The compositions of these luting agents are detailed in Table 1. Each cement was handled according to the manufacturers’ instructions. A transparent Tygon polyethylene mold (Anhui Hongyu Wuzhou Medical Manufacturer; Anhui, China), with an internal diameter of 2.97 mm and a height of 3 mm, was positioned on the treated surface of each specimen and filled completely with the corresponding luting material using a No. 5 explorer to ensure homogeneous distribution. A Mylar strip was placed over the mold and light pressure was applied to stabilize the material. Polymerization was performed for 20 seconds using a VALO Grand light-curing unit (Ultradent; South Jordan, UT, USA) set at an irradiance of 1200 mW/cm². All specimens were then stored in a humid environment at 37°C for 24 hours. Figure 1 illustrates the methodological stages of the specimen preparation process.

**Table 1:** Composition of the materials used

Material	Composition
Variolink Esthetic LC *(Ivoclar Vivadent, Liechtenstein, #2063PL)*	Composed of a polymeric matrix of methacrylate-based resins (Bis-GMA, UDMA, and TEGDMA); inorganic fillers consisting of barium and silica; integrated catalysts; a camphorquinone-based photoinitiator system; and additives such as stabilizers and pigments.
RelyX Veneer *(3M, USA, #10400792)*	Formulated with a polymeric matrix of methacrylate resins (Bis-GMA and TEGDMA); inorganic fillers of silica and fluoroaluminosilicate glass (approximately 63 wt%); organic peroxide-based catalysts; a camphorquinone photoinitiator system; and additional additives including stabilizers and pigments.
Allcem Veneer APS *(FGM, Brazil, #280923)*	Composed of an enhanced polymeric matrix of methacrylate resins (Bis-GMA, UDMA, TEGDMA, and Bis-EMA); inorganic silica fillers treated with silane (approximately 58 wt%); integrated catalysts; an APS (Advanced Polymerization System) photoinitiator; and additives such as stabilizers and rheological modifiers.

### Shear Bond Strength Analysis

After storage, the Tygon molds were carefully removed using a No. 11 surgical blade (Biolife; Sterilance Medical; Suzhou, China). The specimens were examined under magnification to detect voids or defects along the adhesive interface, and any defective samples were excluded. Shear bond strength testing was performed using a universal testing machine (OM150; Odeme Dental Research; Luzerna, Brazil), with a fixture that positioned each cylinder perpendicular to the 1-kN load cell. A chisel-shaped loading device applied shear force at a crosshead speed of 1 mm/min until failure occurred. Bond strength values (MPa) were calculated by dividing the maximum load at failure (N) by the bonded area (mm²). Following testing, each specimen was examined at 10× magnification to classify the failure mode as adhesive (AD), cohesive in the resin cement (CC), cohesive in the milled resin (CR), or mixed (M).

### Statistical Analysis

Data were evaluated for normality using the Shapiro-Wilk test. As the data satisfied the assumptions for parametric testing, one-way ANOVA was performed, followed by Tukey’s post hoc test for pairwise comparisons. A significance level of 5% was adopted for all statistical analyses. The distribution of failure modes was reported descriptively.

## RESULTS

The bond strength values obtained for each cementation protocol are presented in Table 2. The groups luted with RelyX Veneer and Allcem Veneer APS exhibited significantly higher shear bond strength values compared with Variolink Esthetic LC (p < 0.05), while no statistically significant difference was observed between RelyX Veneer and Allcem Veneer APS (p > 0.05). Variolink Esthetic LC demonstrated the lowest mean bond strength among the evaluated protocols. 

**Figure. 2:**
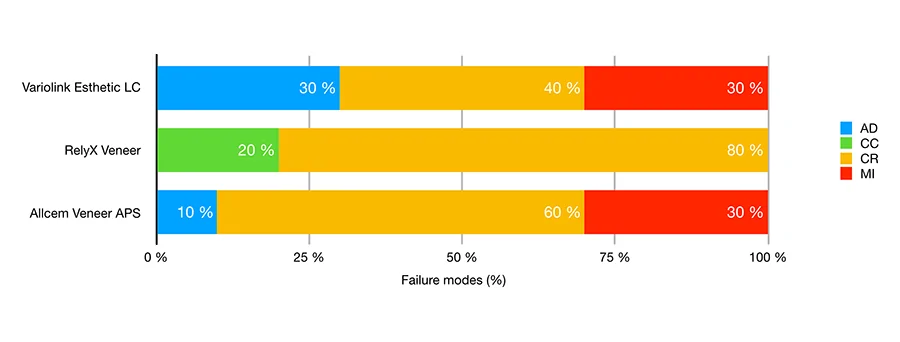
Distribution of failure modes according to the cementation protocol evaluated.

Analysis of failure modes revealed that cohesive failure within the milled resin (CR) predominated across all experimental groups, indicating a relatively strong adhesive interface under the tested conditions. As shown in Figure 2, the Variolink Esthetic LC group displayed a more heterogeneous failure pattern, with 30% adhesive failures (AD), 40% cohesive failures in the resin (CR), and 30% mixed failures (M), which corresponds with its lower bond strength values.

**Table 2:** Mean, standard deviation, and confidence intervals of shear bond strength values (MPa) for the milled resin according to cementation protocol

Protocol	Variolink Esthetic LC	RelyX Veneer	Allcem Veneer APS
Mean	9.24b	10.94a	11.59a
SD	0.55	0.28	0.30
CI	8.90-9.58	10.77-11.11	11.40-11.77

ab Different lowercase letters within the same row indicate statistically significant differences (one-way ANOVA and Tukey’s post hoc test; p < 0.05). SD: standard deviation; CI: confidence interval.

## DISCUSSION

This study demonstrated significant differences in the bond strength of CAD-CAM milled resin blocks depending on the cementation protocol used, underscoring the importance of selecting an appropriate luting agent to optimize clinical outcomes. The light-cured resin cements RelyX Veneer and Allcem Veneer APS achieved the highest bond strength values, reflecting an effective interaction with the Brava Block milled resin, whereas Variolink Esthetic LC exhibited the lowest values, possibly due to compositional differences. Consequently, the null hypothesis was rejected, as statistically significant differences were identified among the evaluated protocols.

From a clinical perspective, the differences observed in adhesive performance are relevant when selecting a luting material for indirect CAD-CAM resin restorations, especially in minimally invasive esthetic procedures-such as veneers and inlays-where retention relies largely on adhesion rather than mechanical retention ^16^. Therefore, the cements that demonstrated superior performance in this study may contribute to greater clinical stability in scenarios where long-term bond durability is critical.

Bond strength between CAD-CAM resin materials and resin cements depends greatly on the adhesive agents employed, as previously highlighted in the literature ^17^. In this context, the protocols using RelyX Veneer and Allcem Veneer APS achieved superior performance compared with the Variolink Esthetic LC protocol, demonstrating an enhanced ability to generate higher bond strength values with the evaluated milled resin. These findings align with prior studies indicating that RelyX Veneer results in lower marginal and internal discrepancies in porcelain veneer cementation ^18^ and provides effective adhesion to CAD-CAM resin materials when combined with surface treatments such as airborne-particle abrasion and silane-based primers ^19^, protocols also used in the present investigation.

The analysis of specific cement properties further supports these findings. Allcem Veneer APS demonstrated lower polymerization shrinkage stress compared with RelyX Veneer ^20^, whereas the latter exhibited high microhardness, indicative of greater resistance to surface deformation ^21^. Both cements have shown excellent adhesion to various substrates ^22^, consistent with the present results. In contrast, Variolink Esthetic LC showed inferior performance, possibly related to reduced adhesive capability despite its higher hardness and flexural modulus. This suggests that individual mechanical properties do not necessarily guarantee optimal adhesive performance, reinforcing the need to evaluate luting agents comprehensively within clinically relevant scenarios ^23^.

The composition of light-cured resin cements also plays a crucial role in their adhesive behavior. RelyX Veneer and Allcem Veneer APS, containing silica-based fillers and advanced polymerization systems, showed better interaction with Brava Block resin compared with Variolink Esthetic LC, which also contains silica but may be limited by factors such as compatibility with specific primers ^24^^,^^25^. Additionally, light-cured cements achieve higher degrees of conversion and improved mechanical properties when light transmission through the restorative material is sufficient ^26^^,^^27^. However, their effectiveness may be reduced in thicker or less translucent restorations, where light attenuation compromises polymerization-an aspect not simulated in this study due to the use of a transparent Teflon mold to ensure uniform exposure.

From a clinical standpoint, the results suggest that combining a standardized surface treatment protocol-aluminum oxide airborne abrasion, ultrasonic cleaning, and silane primer application-with high-performance light-cured cements such as RelyX Veneer or Allcem Veneer APS may promote stronger adhesion between CAD-CAM restorations and tooth structure ^6^^,^^19^. This may reduce the risk of early debonding and enhance clinical predictability. Nevertheless, cement selection must also consider clinical variables such as restoration thickness and translucency, which may influence light transmission and thus the final adhesive performance ^9^^,^^27^.

The predominance of cohesive failures within the milled resin reinforces this interpretation, indicating that under the experimental conditions, the adhesive interface was sufficiently strong for failure to occur within the restorative material itself ^28^. This finding supports the clinical suitability of the evaluated protocols, although direct extrapolation requires consideration of the inherent limitations of in vitro testing.

Finally, for the Variolink Esthetic LC group, the more balanced distribution of adhesive, cohesive, and mixed failures corresponds with its lower bond strength, confirming the material’s sensitivity to cementation conditions.

### Limitations

A major limitation of the present study is the inability to fully reproduce intraoral conditions. The use of a transparent Teflon mold may have facilitated idealized light transmission, without accounting for variations in restoration thickness, translucency, or opacity. Additionally, artificial aging and thermocycling were not performed, although these factors can significantly influence the long-term adhesive performance of resin cements.

Despite these limitations, the study benefits from relevant methodological strengths, including the use of a standardized surface treatment and cementation protocol, rigorous control of experimental variables, and a robust statistical analysis (ANOVA and Tukey), which enabled the detection of true differences among the evaluated systems. These strengths support the reproducibility of the findings and provide a reliable foundation for future investigations that incorporate more complex clinical simulations.

## CONCLUSIONS

Within the limitations of this study, it can be concluded that the cementation protocols using RelyX Veneer and Allcem Veneer APS provided superior bond strength to CAD-CAM milled resin compared with the Variolink Esthetic LC protocol. These findings support the preferential clinical use of high-performance light-cured resin cements for indirect CAD-CAM resin restorations, particularly in minimally invasive adhesive procedures in which the durability of the bond is critical for long-term success.
